# Linear Mixed Effects Models under Inequality Constraints with Applications

**DOI:** 10.1371/journal.pone.0084778

**Published:** 2014-01-21

**Authors:** Laura Farnan, Anastasia Ivanova, Shyamal D. Peddada

**Affiliations:** 1 Lineberger Comprehensive Cancer Center, University of North Carolina, Chapel Hill, North Carolina, United States of America; 2 Department of Biostatistics, University of North Carolina, Chapel Hill, North Carolina, United States of America; 3 Biostatistics Branch, National Institute of Environmental Health Sciences, Research Triangle Park, North Carolina, United States of America; Cleveland Clinic Lerner Research Institute, United States of America

## Abstract

Constraints arise naturally in many scientific experiments/studies such as in, epidemiology, biology, toxicology, etc. and often researchers ignore such information when analyzing their data and use standard methods such as the analysis of variance (ANOVA). Such methods may not only result in a loss of power and efficiency in costs of experimentation but also may result poor interpretation of the data. In this paper we discuss constrained statistical inference in the context of linear mixed effects models that arise naturally in many applications, such as in repeated measurements designs, familial studies and others. We introduce a novel methodology that is broadly applicable for a variety of constraints on the parameters. Since in many applications sample sizes are small and/or the data are not necessarily normally distributed and furthermore error variances need not be homoscedastic (i.e. heterogeneity in the data) we use an empirical best linear unbiased predictor (EBLUP) type residual based bootstrap methodology for deriving critical values of the proposed test. Our simulation studies suggest that the proposed procedure maintains the desired nominal Type I error while competing well with other tests in terms of power. We illustrate the proposed methodology by re-analyzing a clinical trial data on blood mercury level. The methodology introduced in this paper can be easily extended to other settings such as nonlinear and generalized regression models.

## Introduction

**Figure 1:**
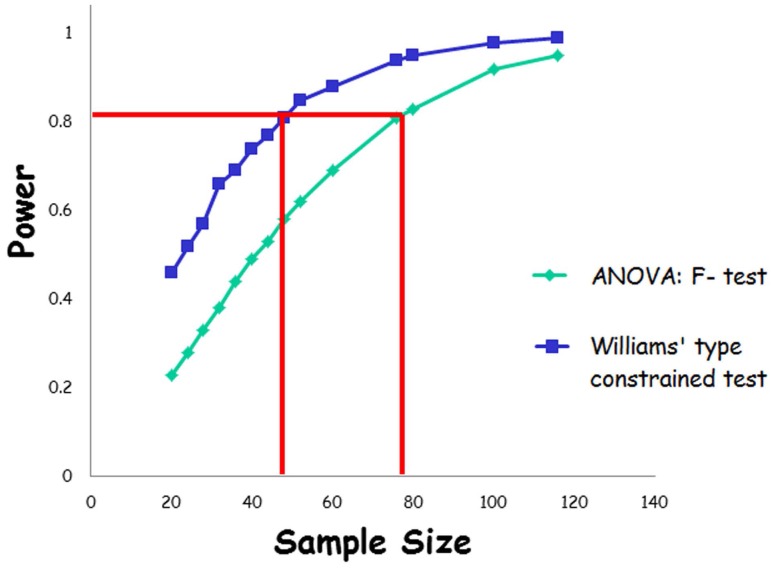
Results of a simulation study to compare power and sample sizes of F-test in One-way ANOVA with the constrained inference Williams’ type test where the critical values are derived using 10,000 bootstrap samples. The power of the Williams test was estimated by averaging 1000 simulated where the critical values are estimated using 10,000 bootstrap samples. The power for F-test was determined using PROC POWER in SAS (9.0). The null hypothesis was that the means of the four dose groups were equal (and zero) and the alternative hypothesis was that the means of the four dose groups have an increasing trend with dose. Data representing the four dose groups were simulated from normal populations with dose means taken to be 0, 0.1, 0.5 and 1, respectively. The actual values of the doses are irrelevant for the two methods described here. The population standard deviation for the four populations was taken to be 1. Corresponding to the 14 different patterns of total sample sizes, namely, 20, 24, 28, 32, 36, 40, 44, 48, 52, 60, 76, 80, 100, 116, the powers of the two methods are plotted. The Type I error was set to 0.05.

In many applications researchers are typically interested in testing for trends or patterns in mean response among two or more experimental or study groups rather than just testing if the groups are different or not. For instance, toxicologists are typically interested in detecting dose-related trends in mean response such as trends in tumor incidence as the dose of a toxin increases [1]–[2]. In cell and circadian biology researchers are often interested in the phase order of genes participating in cell cycle or the circadian clock [3]–[10]. In all such situations researchers are interested in testing for an order among statistical parameters. By performing standard statistical procedures, e.g. the F-test in the case of Euclidean space data, all a researcher can conclude is that there is at least one group which is different from the rest of the experimental groups (in the parameter of interest). Such a conclusion is not very useful if the researcher is interested in proving that the mean response is increasing with dose of the toxin. Furthermore, as noted from the simulation study reported in Figure 1, the power of the standard ANOVA based F-test can be substantially smaller than the power of a test that is designed to detect trend in mean response. For example, note that for a total sample size of 48 (i.e. *n* = 12 per group), the standard ANOVA based F-test yields of power of 0.58 whereas the Williams’ constrained test for trend (which will be described in detail later in this article) yields a power 0.81. Conversely, as also noted in Figure 1, the sample size needed by Williams’ constrained test for a power of approximately 0.80 is 48 whereas the F-test would require a total sample size of 76. This simple motivating example illustrates our point that, in addition to being scientifically relevant, a greater efficiency can be achieved by using statistical methods that take into consideration the investigator’s true hypothesis of interest rather than performing a generic methodology, as often done in scientific literature.

The field of statistics that deals with statistical methods designed to test ordered or constrained hypotheses is commonly called order restricted inference or constrained statistical inference. There exists a very large body of literature on order restricted (constrained inference) spanning nearly sixty years with four books written on the subject, including a recent book by Silvapulle and Sen [11]. Furthermore, for testing for some commonly encountered inequalities in the absence of any covariates in high dimensional data (e.g. gene expression studies), a freely downloadable software called ORIOGEN (Order Restricted Inference for Ordered Gene Expression) (http://www.niehs.nih.gov/research/resources/software/biostatistics/oriogen/) was developed in [12], [13].

In many applications it is common for researchers to be interested in comparing the population means of two or more experimental conditions or groups after adjusting for covariates. Depending upon the study design, as in repeated measurement designs, it is common to use linear mixed effects models to account for the underlying dependence structure as well as the covariates. There exists several decades of literature on statistical inference in linear mixed effects models and numerous books have been written on the subject [14]. The standard statistical test for comparing several population means in a linear mixed effects model framework is the classical *F*-test which is widely used and is available in most standard software packages such as SAS, R and others. However, as noted earlier, by rejecting the null hypothesis using the standard *F*-test one can only infer that at least one population mean is different from the others and hence it may not be ideal for ordered alternatives.

In the absence of any covariates, especially continuous covariates, Mukerjee [15] noted that the usual tests for order restrictions on the means of independent normal populations can be extended to the case when normal populations are correlated as in a repeated measurements design. Later Silvapulle [16] generalized the methodology of [15] to some unbalanced designs with incomplete data. He noted that within-subject correlations make it difficult to generalize some tests into repeated measurement models. Earlier, Singh and Wright [17] considered order-restricted inference on fixed effects in a two-factor mixed model. They presented an analogue to the usual *F*-test for homogeneity and obtained several closed-form results.

It was not until [18]–[20] that statistical inference under inequality constraints in linear mixed effects models was formally addressed. In particular [18] developed an asymptotic likelihood ratio test (LRT) for linear mixed effects model under homoscedastic errors. Since the asymptotic null distribution of LRT depends upon nuisance parameters, they also provided suitable bounds for the distribution using central chi-square distributions with appropriate degrees of freedom. Although these bounds are convenient, our simulation studies suggest that they could potentially be too conservative, especially when the sample sizes are small. Also, the basic assumption made in [18] is that the random effects as well as random errors of the linear model are homoscedastic and normally distributed. In practice this may not necessarily be true. Motivated by the need for a methodology that is robust to non-normality and heteroscedasticity, in Section 2 we provide a general framework using the MINQUE ([14], [21], [22]) and EBLUP type residual based bootstrap [23] to test for any arbitrary linear inequality among means in a linear mixed effects model with possibly heteroscedastic errors.

We present the methodology in Section 2. Results of our simulation study are reported in Section 3. The proposed methodology is illustrated in Section 4 using data from a clinical trial comparing succimer treatment to placebo in children exposed to mercury [24].

## Statistical inference under constraints

### 2.1 The model and notations

Let 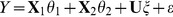
(1)


denote a linear mixed effects models where 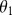 is the vector of treatment effects of the order 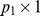, 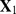 is a design matrix of the order 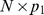 consisting of 0s and 1s, 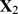 is a known matrix of covariates of the order 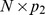 with corresponding (unknown) regression parameter vector 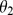 of the order 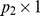, and 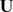 is a 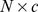 matrix of known design constants. For convenience, we denote 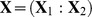 and 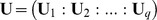, where 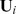 is of order 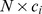, with 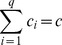, and 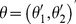 of order 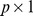, where 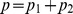. The observation vector 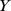 is of the order 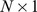 and the unobservable random vectors 
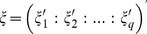
 and 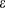 are independently distributed with mean 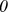 and covariance matrices 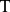 and 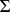, respectively, with 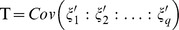 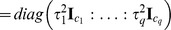. Each 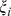, 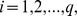 is a random vector of order 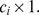 Motivated by applications and for generality we assume a heteroscedastic error structure for 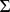, where 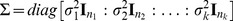 and 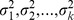 are unknown variances with 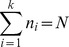.

Let 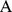 denote a 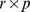 matrix of known constants, such that 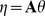 is an 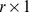 estimable linear function (i.e.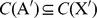, where 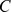 denotes the column space of a matrix). The problem of interest is to test hypotheses of the form:


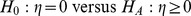
, (2)

where the inequalities are component-wise, with at least one strict inequality. For example, if one is interested in testing a simple order 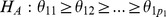 among the components of 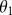, then 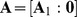 where 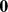 is the null matrix of suitable order and
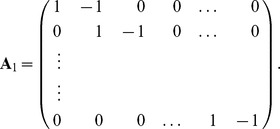



The matrix **A** can be suitably defined in the case of simple tree order 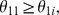 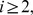 or the umbrella order 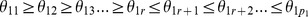, for some *r*, etc.

### 2.2 The likelihood ratio test

Suppose 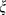 and 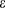 are independently and multivariate normally distributed with log-likelihood function denoted by 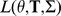. Let (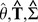) denote the maximum likelihood estimators (MLE) of 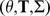 under no constraints and let (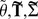) denote the restricted MLE (RMLE) under the alternative hypothesis (2). Then the likelihood ratio test (LRT) statistic is given by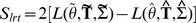. Following the arguments in [18] asymptotically, under the null hypothesis, we obtain the following limiting distribution for LRT:
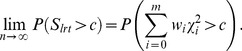
(3)


The weights 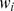 in (3) involve unknown variance components 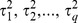 and 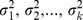. Since in practice one does not know the variance components, one may plug in the estimated values for the unknown variance components in the above limiting distribution [11]. Such plug-in estimators do not perform well unless the sample size is very large. Furthermore, as the number of treatment groups increases, the computation of the weights 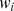 in (3) is a challenging problem. Recognizing this challenge, simple central chi-square distribution based bounds for the limiting probability in (3) were derived in [18]. These probabilities can be used for deriving bounds for the asymptotic p-values.

### 2.3 The residual bootstrap based test

**Figure 2:**
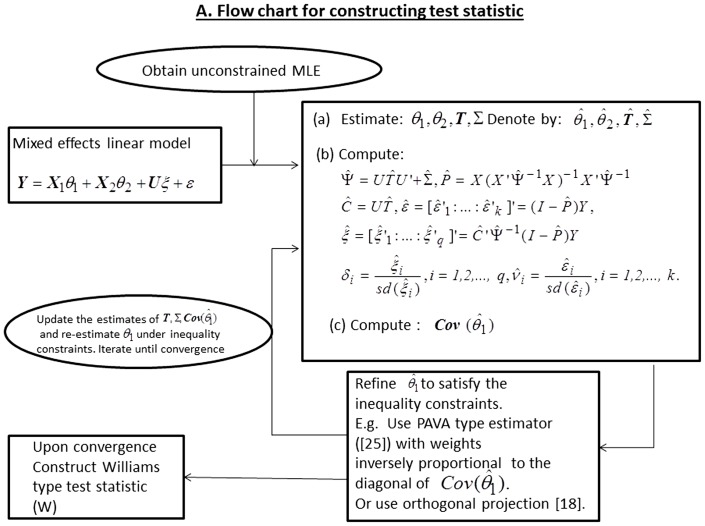
Flow chart for constructing test statistic.

There are three differences between our approach and the above LRT approach. Firstly, rather than using the RMLE which, at each step of the iteration, projects the unconstrained estimator 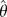
onto the set of constraints in the alternative hypothesis, we use the algorithm described in [25]. The estimation algorithm in [25] is identical to the pool adjacent violators algorithm (PAVA) when the constraint is a simple order, otherwise it is a modification of PAVA. Our choice of PAVA is motivated by the fact that RMLE can potential fail under some conditions [25]. Briefly, the PAVA is implemented as follows. Consider a sequence of three numbers 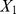 **,** 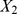 and 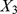, and suppose in theory these numbers are expected to be non-decreasing. However, suppose the observed values are 3, 1 and 4, respectively. The expected order is violated by the first two numbers and therefore the PAVA averages the two violators, resulting in the ordered numbers 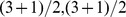 and 4. Depending upon the situation, one could use weighted averages rather than simple average. As noted in the flow chart provided in Figure 2, our constrained estimation algorithm is flexible as one can replace PAVA type estimator [25] by the orthogonal projection estimator along the lines of [18]. Details of our algorithm (Algorithm A.1) are provided in the Appendix S1. Secondly, as noted above, because the asymptotic distribution of the LRT is non-trivial to use in practice we use a computationally simple non-parametric bootstrap to derive the p-values. Lastly, as an alternative to LRT statistic we use a Williams’ type test statistic [26]–[28]. Intuitively, the Williams’ test for monotonic order is a generalization to the idea of standard t-test with the exception that the numerator is the difference between the estimates of the largest parameter and smallest parameter, where the estimates are obtained by using the PAVA.

Let 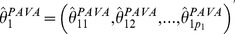 denote the PAVA type constrained estimator derived according to the algorithm proposed in this paper (Figure 2). Then as in [29] we may define Williams’ type test statistic [26]–[28] for various order restrictions. In the following we provide the test statistic for three commonly encountered order restrictions. In each case the null hypothesis is 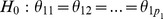. For the others one may appeal to the general framework provided in [26].

#### Simple order (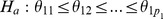)


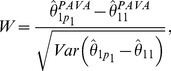
(4)

where 
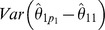
 is the estimated variance of the contrast 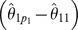.

#### Simple tree order (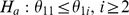)



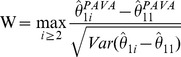
(5)


where 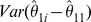 is the estimated variance of the contrast 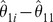.

#### Umbrella order (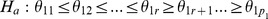)



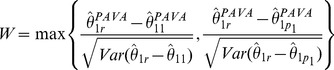
(6)


where 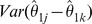 is the estimated variance of the contrast 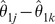.

**Figure 3:**
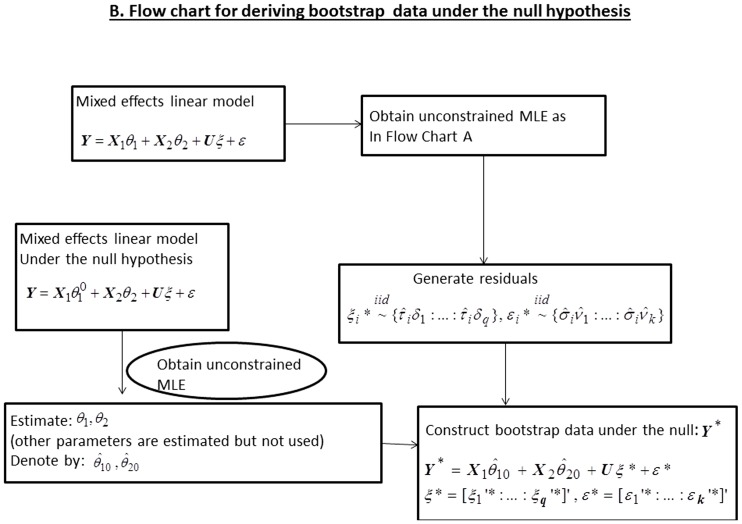
Flow chart for deriving Bootstrap data under the null hypothesis.

The bootstrap methodology to obtain the p-values is described in the flow chart in Figure 3 and the details are provided in Algorithm A.2 in the Appendix S1. Although in this paper we are using the Williams’ type statistic *W*, one may use a likelihood ratio type statistic (or any other constrained test statistic of user’s choice) instead of *W* but use residual bootstrap described in this paper for deriving the p-values. Such a strategy may result in a better power for some patterns of the mean parameters. Thus the framework developed in this paper is fairly flexible.

## Simulation study

### 3.1 Study design

We evaluated the Type I error and power of the proposed EBLUP bootstrap test for the case of simple order using the proposed statistic (4). We compared our method with the asymptotic likelihood ratio test [18] using the upper bound in the equation (4.6) of [18] for deriving the critical values. We considered a variety of patterns of parameters and covariance matrices as follows:

#### Normally distributed data

The data were simulated using model (1) with the number of subjects per each treatment 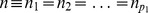 **,** 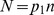, 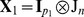 **, a** 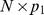 design matrix consisting of 0’s and 1’s, 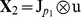, where *u* was generated as a 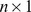 vector, with its components uniformly distributed in [0, 2], 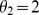, a corresponding regression parameter, 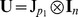, 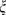, a 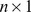 vector of independent subject random effects, and 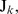 a 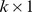 vector of 1s. The random vectors 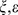 were independently and normally distributed with means 0 and covariance matrices 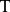 and 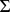, where 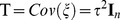. Simulations were performed for 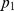 = 3, 5 treatment groups, 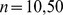 subjects per each treatment and five different patterns of treatment means 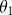: (P1) (0,0,..., 0), (P2) (0,..., 0, *a*), (P3) (0, *a*,..., *a*), (P4) (*a*, 2*a*,..., 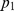 *a*), (P5) (0, *a*,..., *a*, 2*a*).

Here different values of *a* in the interval [0, 2] were chosen arbitrarily to get a sense of power for a variety of patterns. Note that pattern (P1) corresponds to the null pattern for computing Type I error. Patterns (P2) to (P5) are patterns of 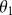 where the components satisfy a simple order constraint 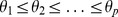 for evaluating the power of the two test procedures. Two different structures of 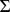 were considered, namely, homoscedastic error structure with 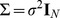 and heteroscedastic error structure with 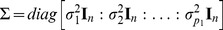, where 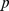 is the number of treatments, and 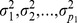 are unknown variances with 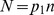. The patterns of 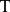 and 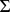 considered in our simulations are as follows:

- *Homoscedastic case:* We fixed 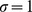 and considered three patterns of 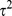, namely 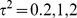.
- *Heteroscedastic case:* We fixed 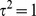 and, as commonly done, we chose the error variance to be a function of the mean, namely 
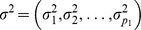
, where 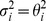. In the case of null hypothesis, i.e., 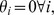 we chose 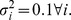

#### Log-normally distributed data

All results are based on 500 simulation runs. We used 500 bootstrap runs to generate the null distribution for the proposed bootstrap test. In all simulations the nominal value for Type I error was taken to be 0.05.

### 3.2 Results

Complete simulation scenarios and results are presented in Tables 1–6. Both tests operate at the desired nominal Type I error rate, although sometimes the asymptotic likelihood ratio test tends to be slightly conservative. Generally the proposed test is substantially more powerful than the asymptotic likelihood ratio test. In some cases the gains in power are as much as 0.36, e.g. 0.78 vs. 0.42 for heteroscedastic normally distributed data with 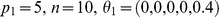.

**Table 1:** Type I errors for homoscedastic normally distributed data.

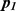	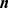	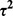	Asymp-LRT	Proposed method
3	10	1	0.03	0.05
3	50	1	0.01	0.03
3	10	0.2	0.05	0.04
3	10	2	0.04	0.05
3	50	2	0.01	0.03
5	10	1	0.02	0.03
5	10	0.2	0.02	0.04
5	10	2	0.02	0.04

**Table 2 pone-0084778-t002:** Power for homoscedastic normally distributed data.

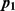	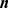	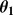										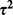	Asymp-LRT	Proposed method
3	10	0	0.00	1.25								1	0.82	0.84
3	10	0	1.26	1.26								1	0.82	0.84
3	50	0	0.55	0.55								1	0.85	0.89
3	10	0	0.73	1.45								1	0.86	0.89
5	10	0	0.00	0.00	0.00	1.27						1	0.65	0.86
5	10	0	1.24	1.24	1.24	1.24						1	0.58	0.86
5	10	0	0.37	0.74	1.11	1.48						1	0.80	0.90
5	10	0	0.81	0.81	0.81	1.62						1	0.74	0.93

**Table 3 pone-0084778-t003:** Type I errors for heteroscedastic normally distributed data.

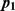	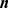	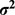					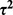	Asymp-LRT	Proposed method
3	10	0.1	0.10	2.37			1	0.04	0.03
3	10	0.1	0.20	0.20			1	0.03	0.04
3	10	0.1	0.09	0.36			1	0.03	0.03
3	50	0.1	0.10	0.01			1	0.01	0.03
3	50	0.1	0.02	0.02			1	0.02	0.04
5	10	0.1	0.10	0.10	0.10	0.16	1	0.01	0.04
5	10	0.1	0.20	0.20	0.20	0.20	1	0.01	0.04
5	10	0.1	0.11	0.44	0.99	1.76	1	0.01	0.04
5	10	0.1	0.11	0.11	0.11	0.45	1	0.02	0.05

**Table 4 pone-0084778-t004:** Power for heteroscedastic normally distributed data.

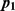	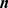	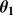					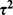	Asymp-LRT	Proposed method
3	10	0	0.00	1.54			1	0.82	0.88
3	10	0	0.45	0.45			1	0.81	0.82
3	10	0	0.30	0.60			1	0.82	0.80
3	50	0	0.00	0.10			1	0.70	0.74
3	50	0	0.15	0.15			1	0.86	0.92
3	50	0	0.08	0.16			1	0.95	0.93
5	10	0	0.00	0.00	0.00	0.40	1	0.42	0.78
5	10	0	0.45	0.45	0.45	0.45	1	0.68	0.81
5	10	0	0.33	0.66	1.00	1.33	1	0.96	0.88
5	10	0	0.34	0.34	0.34	0.67	1	0.71	0.82

**Table 5 pone-0084778-t005:** Type I errors for log-normally distributed data.

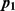	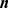	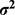					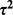	Asymp-LRT	Proposed method
3	10	0.10	0.10	0.04			1	0.02	0.03
3	10	0.10	0.20	0.20			1	0.03	0.05
3	10	0.10	0.01	0.04			1	0.03	0.03
3	50	0.10	0.02	0.02			1	0.01	0.01
3	50	0.10	0.01	0.03			1	0.01	0.01
5	10	0.10	0.10	0.10	0.10	0.16	1	0.02	0.04
5	10	0.10	0.04	0.04	0.04	0.04	1	0.02	0.03
5	10	0.10	0.01	0.04	0.09	0.16	1	0.02	0.05
5	10	0.10	0.01	0.01	0.01	0.04	1	0.03	0.03

**Table 6:** Power for log-normally distributed data.

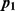	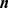	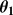					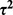	Asymp-LRT	Proposed method
3	10	0	0.00	1.54			1	0.90	0.98
3	10	0	0.45	0.45			1	0.81	0.85
3	10	0	0.30	0.60			1	0.85	0.90
3	50	0	0.00	0.20			1	0.92	0.88
3	50	0	0.15	0.15			1	0.57	0.68
3	50	0	0.08	0.16			1	0.89	0.69
5	10	0	0.00	0.00	0.00	0.40	1	0.44	0.77
5	10	0	0.45	0.45	0.45	0.45	1	0.66	0.83
5	10	0	0.10	0.20	0.30	0.40	1	0.79	0.75
5	10	0	0.34	0.34	0.34	0.67	1	0.72	0.94
5	50	0	0.00	0.00	0.00	0.20	1	0.97	0.96
5	50	0	0.20	0.20	0.20	0.20	1	0.66	0.92
5	50	0	0.08	0.08	0.08	0.16	1	0.87	0.74

## Illustration

**Figure 4:**
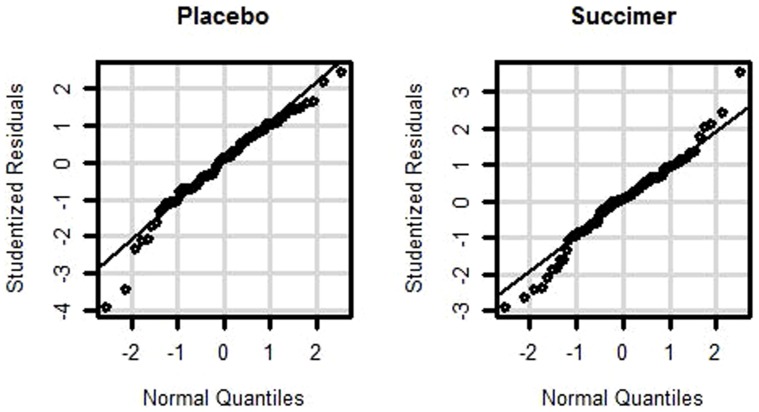
Normal quantile-quantile plots of studentized residuals from regressing log organic mercury in the placebo and succimer groups.

We illustrate the proposed methodology using data from patients in a randomized placebo-controlled, double-blind clinical trial the Treatment of Lead-exposed Children trial, or TLC [30]. In TLC, 384 children aged 12–33 months were assigned to the placebo group and 396 to the succimer group. In the succimer group, up to three 26-day courses were administered. Cao et al. [24] measured and analyzed mercury levels in pre-treatment to investigate whether succimer, a mercaptan compound known to reduce blood lead concentration in children, also reduces blood mercury concentration. At the baseline, blood mercury levels were quantified in 657 samples (338 succimer and 319 placebo). At 1-week post treatment, total mercury concentration was quantified in 623 samples (313 succimer and 310 placebo). After 5 months of treatment, blood mercury levels were quantified in 61 samples: 30 succimer treated children and 31 placebo treated children. To investigate the efficacy of succimer treatment relative to placebo, Cao et al. [24] tested for an increasing trend over time in the difference between the mean mercury concentrations in the succimer group and the placebo group, after adjusting for child’s age, sex, race and the study center. The authors concluded that there was no significant trend in the mean difference in mercury concentrations in the succimer group and the placebo group. Since their analysis ignored the covariance structure induced by repeated measurements, we reanalyzed the data using the proposed methodology which accounts for the repeated measurements in the data. We fitted a linear mixed effects model with log organic mercury as a dependent variable, treatment, race, gender, center and age as fixed effects, and subject as a random effect. The normal quantile-quantile plots (Figure 4) of resulting studentized residuals in the placebo and succimer groups suggest that the data are potentially non-normally distributed.

Denoting the difference in the mean log mercury levels at the *t*th time between placebo and succimer groups as by 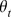 (*t* = 1 for baseline, *t* = 2 for 1-week and *t* = 3 for 5-months) we tested the following hypothesis:
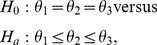



with at least one strict inequality. The three UMLEs were 0.59, 0.92, 0.73 and the MINQUE-based constrained estimates were 0.59, 0.82, 0.82. Using the proposed test we obtained a bootstrap p-value of 0.109. Thus, we are not able to reject the null hypothesis in favor of a trend in the difference in means. These results are consistent with conclusions of [24], that succimer chelation for low level organic mercury exposure in children has limited efficacy.

## Summary and Concluding Remarks

Inequality constraints arise naturally in many applications, such as toxicology, where researchers are interested in studying dose-response of a chemical, or gene expression studies in oncology, where a researcher may be interested in understanding the changes in gene expression according to cancer stage. We proposed a new method to test for inequality constraints. Since the method uses Rao’s MINQUE theory (cf. [22]) for estimating variance components and PAVA for estimating the means, it does not necessarily require normality. In the simple order restriction, our extensive simulation studies suggest that the proposed methodology provides a better control of type I error than the asymptotic likelihood ratio test of [18] when the data are non-normally distributed. Our proposed methodology seems to control the Type I error at the desired nominal level.

In the manuscript we considered one-dimensional order constraints. In toxicology, researchers are often interested in dose 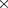 time response surfaces, which results in a two-way classification that can be expressed as a constrained inference problem with constraints on rows and columns of a matrix. The proposed testing procedure can be easily extended to deal with such cases.

## Supporting Information

Appendix S1 **Description of Algorithms A.1 and A.2.**
(DOCX)
