# Supplementary material for: Linear Mixed Effects Models under Inequality Constraints with Applications
Source: PLoS One. 2014 Jan 21;9(1):e84778. doi: 10.1371/journal.pone.0084778 (PMC3897384; doi:10.1371/journal.pone.0084778)
Supplement: Appendix S1 — Description of Algorithms A.1 and A.2. (DOCX) [file pone.0084778.s001.docx]

# Appendix S1: Description of Algorithms A.1 and A.2.

*Algorithm A.1. An E-M type algorithm for constrained estimation in linear mixed models*

As noted earlier in this article, our testing procedure is flexible as one could any constrained estimator (e.g. RMLE). Along the lines of [1] we propose a simple E-M type algorithm to obtain a constrained estimator as described below. If the unconstrained estimator of $\theta$ is either exactly or asymptotically multivariate normally distributed then following arguments in [2], under some suitable conditions, the resulting constrained estimator of $\eta=\mathbf{A}\theta$ would converge to the restricted maximum likelihood estimator. Thus, in the following we do not assume that the underlying data are normally distributed. However, since we are using the MINQUE for the variance components, the unconstrained estimator of $\theta$ (under suitable regularity conditions) is asymptotically normally distributed.

Let$\hat{\theta}_{1}^{(m)}$,$\hat{\theta}_{2}^{(m)}$, $\hat{\tau}^{2(m)}$, $\hat{\sigma}^{2(m)}$ denote the *m*th iterate estimates of $\theta_{1}$,$\theta_{2}$, $\tau^{2}$, and $\sigma^{2}$, respectively.

*Step 0* (*m =* 0). Let$\mathbf{X}=\left[\mathbf{X}_{1}:\mathbf{X}_{2}\right]$, $\theta=\left(\theta'_{1},\theta'_{2}\right)^{'}$. Compute$\theta^{(0)}=\left(\mathbf{X}'\mathbf{X}\right)^{-1}\mathbf{X}'Y$, the ordinary least squares estimator for $\theta$. Compute $\sigma_{i}^{2(0)} =\frac{1}{n_{i}}\left\|Y_{i}-\mathbf{X}_{i}\theta^{(0)}\right\|^{2}\text{ for }i=1,\ldots,k.$ For $\hat{\tau}_{i}^{2(0)}$ we use MINQUE [3].

*Step 1*. Set *m* = *m* + 1. Fix $\theta_{1}$, $\theta_{2}$, and $\tau^{2}$ at $\hat{\theta}_{1}^{(m-1)}$, $\hat{\theta}_{2}^{(m-1)}$, $\hat{\tau}^{2(m-1)}$ respectively, and iteratively estimate $\sigma^{2}$ (for *m* = 1, 2, …):

$\begin{matrix} \hat{\sigma}_{i}^{2(m)} =\sigma_{i}^{2(m-1)} +\frac{1}{n_{i}}\hat{\sigma}_{i}^{4(m-1)}\times\\ tr\left[\left(\hat{\mathbf{\Psi}}^{(m-1)}\right)^{-1}\left(Y-\mathbf{X}_{1}\hat{\theta}_{1}^{(m-1)}-\mathbf{X}_{2}\hat{\theta}_{2}^{(m-1)}\right)\left(Y-\mathbf{X}_{1}\hat{\theta}_{1}^{(m-1)}-\mathbf{X}_{2}\hat{\theta}_{2}^{(m-1)}\right)^{'}\left(\hat{\mathbf{\Psi}}^{(m-1)}\right)^{-1}\right. \\ \left.-\left(\hat{\mathbf{\Psi}}^{(m-1)}\right)^{-1}\right]_{ii},\text{ }i=1,\ldots,k, \end{matrix}$ (A1)

where $\hat{\mathbf{\Psi}}^{(m-1)}=\mathbf{U}\hat{\mathbf{T}}^{(m-1)}\mathbf{U}'+\hat{\mathbf{\Sigma}}$, $\hat{\mathbf{T}}^{(m-1)}=diag\left(\hat{\tau}_{1}^{2(m-1)}\mathbf{I}_{c_{1}}:\ldots:\hat{\tau}_{q}^{2(m-1)}\mathbf{I}_{c_{q}}\right)$, $\hat{\sigma}^{2(0)}=\sigma^{2(m-1)}$, and **A***_ii_* indicates the (*i*, *i*)th block of **A**.

*Step 2*.

(a) Fix $\sigma^{2}$ at $\hat{\sigma}^{2(m-1)}$ and iteratively estimate $\theta_{1}$, $\theta_{2}$, and $\tau^{2}$ using the following equations (for *m* = 1, 2, …):

$\hat{\theta}_{1}^{(m)}=\hat{\theta}_{1}^{(m-1)}+\left(\mathbf{X}'_{1}\hat{\mathbf{\Sigma}}^{-1}\mathbf{X}_{1}\right)^{-1}\mathbf{X}'_{1}\left(\hat{\mathbf{\Psi}}^{(m-1)}\right)^{-1}\left(Y-\mathbf{X}_{1}\hat{\theta}_{1}^{(m-1)}-\mathbf{X}_{2}\hat{\theta}_{2}^{(m-1)}\right)$, (A2)

$\hat{\theta}_{2}^{(m)}=\hat{\theta}_{2}^{(m-1)}+\left(\mathbf{X}'_{2}\hat{\mathbf{\Sigma}}^{-1}\mathbf{X}_{2}\right)^{-1}\mathbf{X}'_{2}\left(\hat{\mathbf{\Psi}}^{(m-1)}\right)^{-1}\left(Y-\mathbf{X}_{1}\hat{\theta}_{1}^{(m-1)}-\mathbf{X}_{2}\hat{\theta}_{2}^{(m-1)}\right),$ (A3)$\begin{matrix} \hat{\tau}_{i}^{2(m)} =\hat{\tau}_{i}^{2(m-1)} +\frac{\hat{\tau}_{i}^{4(m-1)}}{c_{i}}tr\left\{\mathbf{U}' _{i} \left[\left(\hat{\mathbf{\Psi}}^{(m-1)}\right)^{-1}\left(Y-\mathbf{X}_{1}\hat{\theta}_{1}^{(m-1)}-\mathbf{X}_{2}\hat{\theta}_{2}^{(m-1)}\right)\right.\right.\times\\ \times\left(Y-\mathbf{X}_{1}\hat{\theta}_{1}^{(m-1)}-\mathbf{X}_{2}\hat{\theta}_{2}^{(m-1)}\right)^{'}\left(\hat{\mathbf{\Psi}}^{(m-1)}\right)^{-1}\left. -\left.\left(\hat{\mathbf{\Psi}}^{(m-1)}\right)^{-1}\right]\mathbf{U}_{i}\right\}\text{, }i=1,\ldots,q, \end{matrix}$ (A4)

where$\hat{\theta}^{(0)}=\theta^{(m-1)}$, $\hat{\tau}^{2(0)}=\tau^{2(m-1)}$, $\hat{\mathbf{\Psi}}^{(m-1)}=\mathbf{U}\hat{\mathbf{T}}^{(m-1)}\mathbf{U}'+\hat{\mathbf{\Sigma}}$, and $\hat{\mathbf{T}}^{(m-1)}$ is$diag\left(\tau_{1}^{2(m-1)}\mathbf{I}_{c_{1}}:\ldots:\tau_{q}^{2(m-1)}\mathbf{I}_{c_{q}}\right)$. Note that although we could have combined equations (A2) and (A3) and use $(\mathbf{X}'\mathbf{\Sigma}^{-1}\mathbf{X})\mathbf{X}'\mathbf{\Sigma}^{-1}\mathbf{Y}$ to obtain a combined updated estimate for , our proposed approach is computationally more stable and simpler.

(b) Using the estimates obtained in (a), we apply the PAVA type methodology along the lines of [4] with weights proportional to the inverse of the sample sizes to obtain $\hat{\theta}_{1}^{(m)}$ under the desired inequality constraints. Alternatively, one could perform the following constrained optimization to obtain $\hat{\theta}_{1}^{(m)}$:

$\min_{\mathbf{A}\theta\geq0} (Y-\mathbf{X}_{1}\theta_{1}^{i}-\mathbf{X}_{2}\hat{\theta}_{2}^{i})'\hat{\mathbf{\Psi}}^{-1(i)}(Y-\mathbf{X}_{1}\theta_{1}^{i}-\mathbf{X}_{2}\hat{\theta}_{2}^{i})$. (A5)

*Steps 1 and 2* are iterated until convergence. The resulting constrained estimators are denoted by $\hat{\theta}=\left(\hat{\theta}'_{1}\text{,}\hat{\theta'}_{2}\right)^{'}$. Since it is well-known that the RMLE can perform poorly, even for simple order restriction when the data are correlated (see [4]), therefore as an alternative to (A5), we consider PAVA type methodology of [4]. Thus the point estimators derived here are very generally applicable for any order restriction. In Step 2(b), if one uses (A5) for obtaining a constrained estimator then applying the general theory established in [5], we note that $\hat{\theta}=\left(\hat{\theta'}_{1}\text{,}\hat{\theta'}_{2}\right)^{'}$ is consistent.

To describe the MINQUE methodology for estimating we rewrite the linear model (1) as $Y=\mathbf{X}\theta+\zeta$, where $\theta=\left(\theta'_{1},\theta'_{2}\right)^{'}$, $\mathbf{X}=\left(\mathbf{X}_{1}:\mathbf{X}_{2}\right)$ with

$E(\zeta)=0$, , $\mathbf{F}_{i}=\mathbf{U}_{i}\mathbf{U}'_{i},\text{ }i=1,2,\text{ }...,q,$ and $\mathbf{F}_{i}=Diag[0:0:...:\mathbf{I}:0...0]$, $i=q+1,2,\text{ }...,q+k$ with the identity matrix of order $n_{i-q}\times n_{i-q}$ located at the *i*th location. Each $\mathbf{F}_{i}$ is $N\times N$. Let $\mathbf{F}=\left[\mathbf{F}_{1}:\mathbf{F}_{2}:...:\mathbf{F}_{\text{q}+\text{k}}\right]$, , , , and .

In the above expression, $\mathbf{A}^{-}$ denotes a *generalized inverse* (or *g-inverse*) of $\mathbf{A}$. The above expressions are invariant to the choice of g-inverse. Hence without loss of generality one may use the Moore-Penrose inverse$\mathbf{A}^{+}$. From [3], the MINQUE of is then obtained by solving the system of linear equations where denotes an initial estimate of . Since the MINQUE depends upon the initial estimate, Rao and Kleffe [6] recommend iterating as above until convergence. The resulting estimator is known as the iterated MINQUE (or I-MINQUE). Denote the I-MINQUE of by . As discussed in [7], estimated parameters can be negative. As is commonly done, in such cases we replace them with 0.01. Since $\eta=\mathbf{A}\theta$ is an estimable linear function of $\theta$, its weighted least squares estimator is given by .

Let

R1: .

R2: $E\left\|\zeta\right\|^{4}<\infty$.

R3: , where the matrix $\mathbf{D}=\left(d_{ij},\text{ }i,j=1,2,\ldots,q+k\right)$ is non-singular.

R4: , where $\lambda_{\max}\left(\mathbf{H}\right)$ is the largest eigenvalue of a matrix $\mathbf{H}$.

R5: $(X'W_{\varphi}^{+}X)^{+}\to M(\varphi)$, where is a positive definite matrix.

R6: .

**Theorem A1**:

For any estimable linear function $\mathbf{A}\theta$ in a linear mixed model (1) satisfying the regularity conditions R1 to R6,

$$A(\hat{\theta}-\theta\text{) } \sim^{asymptotically} \text{ }N(0,AM(\varphi)^{-1}A')$$

**Proof**: Under the regularity conditions R1 to R4, from Theorem 10.2.3 in [6] we deduce that the MINQUE is consistent for . Appealing to Noether’s conditions (R5 and R6) we deduce the asymptotic normality of $\mathbf{A}\hat{\theta}$ from the discussion in Chapter 10.7 in [6].

*Algorithm A.2. The EBLUP based bootstrap methodology*

To derive the p-values of the above test statistics, we now describe the non-parametric EBLUP bootstrap methodology for deriving the null distribution of the test statistic (9). We begin by constructing bootstrap sample $Y^{*}$ as follows.

*Step 1:* Obtain the point estimator of $\theta=\left(\theta'_{1},\theta'_{2}\right)^{'}$ under the null hypothesis. Denote it by.

*Step 2:* Let $\hat{\theta}=\left(\hat{\theta}'_{1},\hat{\theta}'_{2}\right)^{'}$,$\hat{\mathbf{T}}$, and $\hat{\mathbf{\Sigma}}$ denote the unconstrained point estimators of $\theta=\left(\theta'_{1},\theta'_{2}\right)^{'}$, $\mathbf{T}$and $\mathbf{\Sigma}$. Compute

$$\begin{matrix} \hat{\mathbf{C}}=\mathbf{U}\hat{\mathbf{T}},\text{ }\hat{\mathbf{\Psi}}=\mathbf{U}\hat{\mathbf{T}}\mathbf{U}'+\hat{\mathbf{\Sigma}},\text{ }\hat{\mathbf{P}}=\mathbf{X}(\mathbf{X}'\hat{\mathbf{\Psi}}^{-1}\mathbf{X})^{-1}\mathbf{X}'\hat{\mathbf{\Psi}}^{-1},\text{ } \\ \hat{\varepsilon}=(\mathbf{I}-\hat{\mathbf{P}})Y,\hat{\xi}=\left(\hat{\xi}'_{1}:\hat{\xi}'_{2}:...:\hat{\xi}'_{q}\right)^{'}=\hat{\mathbf{C}}'\hat{\mathbf{\Psi}}^{-1}(\mathbf{I}-\hat{\mathbf{P}})Y. \end{matrix}$$

*Step 3:* Let $\hat{\delta}_{i}=\frac{\hat{\xi}_{i}}{sd(\hat{\xi}_{i})},i=1,2,...,q$ and let $\hat{\nu}_{i}=\frac{\hat{\varepsilon}_{i}}{sd(\hat{\varepsilon}_{i})},i=1,2,...,k$ where $sd(.)$ represents the usual sample standard deviation of the elements in the vector.

*Step 4*: Let $\hat{\delta}_{i}^{*},i=1,2,...,q$, denote a random vector obtained by taking a random sample (with replacement) of size $c_{i}\times1,\text{ }i=1,2,\ldots,q$ from the components of $\hat{\delta}_{i}$. Similarly, let$\hat{\nu}_{i}^{*}$, $i=1,2,\ldots,k$ denote a random vector obtained by taking a random sample (with replacement) of size $n_{i}\times1$ from the components of $\hat{\nu}_{i}$. Finally, let $\hat{\xi}^{*}=\hat{\tau}_{i}\hat{\delta}_{i}^{*}$, $i=1,2,\ldots,q$ and $\hat{\varepsilon}_{i}^{*}=\hat{\sigma}_{i}\hat{\nu}_{i}^{*}$, $i=1,2,\ldots,k$, then the EBLUP bootstrap sample $Y^{*}$ is constructed using the following equation

$$Y^{*}=\mathbf{X}\tilde{\theta}^{0}+\mathbf{U}\hat{\xi}^{*}+\hat{\varepsilon}^{*}.$$

The above model honors the null hypothesis regarding the parameter $\theta=\left(\theta'_{1},\theta'_{2}\right)^{'}$ as well as honors the underlying variance components structure.

REFERENCES

1 Hoferkamp CL, Peddada SD (2002). Parameter estimation in linear models with heteroscedastic variances subject to order restrictions. Journal of Multivariate Analysis 82: 65-87.

2 Davidov O, Rosen S (2011). Constrained inference in mixed-effects models for longitudinal data with application to hearing loss. Biostatistics 12: 327-340.

3 Rao CR (1972). Estimation of variance and covariance components in linear models. Journal of the American Statistical Association 67: 112-115.

4 Hwang JTG and Peddada SD (1994). Confidence Interval Estimation Subject to Order Restrictions. Annals of Statistics, 22: 67-93.

5 Nettleton, D (1999). Convergence properties of the em algorithm in constrained parameter spaces. The Canadian Journal of Statistics / La Revue Canadienne de Statistique 27: 639-648.

6 Rao CR, Kleffe J (1988). Estimation of variance components and applications.

Amsterdam; New York; New York, N.Y., U.S.A.: North-Holland.

7 Rao JNK, Subrahmaniam K (1971). Combining independent estimators and

estimation in linear regression with unequal variances. Biometrics 27: 971-990.
